# Delineating the dyadic coping process in HIV serodiscordant male couples: a dyadic daily diary study using the common fate model

**DOI:** 10.1007/s11136-025-03903-4

**Published:** 2025-02-03

**Authors:** Jianhua Hou, Rong Fu, Taiyi Jiang, Nancy Xiaonan Yu

**Affiliations:** 1 Department of Social and Behavioural Sciences, City University of Hong Kong, Hong Kong, People’s Republic of China; 2 Beijing Key Laboratory for HIV/AIDS Research, Clinical and Research Center for Infectious Diseases, Beijing Youan Hospital, Capital Medical University, Beijing, People’s Republic of China

**Keywords:** Common fate model, Couples, Daily diary, Dyadic coping, HIV

## Abstract

**Purpose:**

Although HIV is likely to be a couple-based issue among serodiscordant male couples due to cross-partner transmission, little is known about how they cope with HIV as a team. This study aimed to examine the dyadic coping process among serodiscordant couples.

**Methods:**

A dyadic daily diary study was used to answer our research questions. One hundred and forty-one Chinese HIV serodiscordant male couples completed measures of we-disease appraisal, common dyadic coping, quality of life, and relationship satisfaction for 14 consecutive days and ART adherence, attitudes toward PrEP, quality of life, and relationship satisfaction at a 2-month post-diary assessment. Computations were based on the common fate mediation model, using multilevel structural equation modeling.

**Results:**

Regarding direct effect, we-disease appraisal was associated with both partners’ quality of life at the between-person level, post-diary quality of life, attitudes toward to PrEP, relationship satisfaction at both levels as well as post-diary relationship satisfaction. Common dyadic coping mediated the association between we-disease appraisal and both partners’ quality of life as well as relationship satisfaction at the within-person level. However, no mediating effects were observed at the between-person level.

**Conclusions:**

Our findings highlighted the importance of the daily dyadic coping process among HIV serodiscordant couples. Future interventions should direct resources toward fostering a shared disease appraisal and training couples in common dyadic coping strategies for their daily lives.

**Supplementary Information:**

The online version contains supplementary material available at 10.1007/s11136-025-03903-4.

In male serodiscordant couples, with one partner living with HIV (PLWH) while the other is not, the risk of HIV infection is heightened [1]. For instance, the estimated prevalence of such cases rose from 34 to 40% between 2002 and 2010 [2]. HIV serodiscordant male couples may face unique relational, sexual, and social challenges. These may include disclosing their HIV status, managing fears of HIV transmission, negotiating sexual practices, and coping with stigma and discrimination [3, 4]. Studies on HIV serodiscordancy have shown that living with HIV can compromise quality of life [5, 6] and relationship satisfaction [7, 8].

A prominent framework helpful for understanding the significance of romantic relationships is *dyadic coping*. Bodenmann [9] initially defined dyadic coping as “all efforts of one or both partners to face and manage stress events as well as strains affecting one of the partners or both together.” Berg and Upchurch [10] and Badr and Acitelli [11] later expanded this definition to the chronic illness contexts and collectively emphasize two core elements of the dyadic coping process: a) dyadic disease appraisal and b) dyadic coping behaviors. While dyadic coping can occur in any established relationship, the present study focuses specifically on the dyadic coping process in serodiscordant male couples.

## We-disease appraisal

We-disease appraisal refers to viewing the illness as a shared experience within the relationship [10]. Berg and Upchurch [10] proposed that viewing the illness as “ours” rather than “mine” or “yours” can optimize the couple’s ability to jointly manage illness and lead to better outcomes. To date, most studies have treated we-disease appraisal as a characteristic of the couple, with couples categorized as appraising the illness as the patient’s alone or as shared within a romantic relationship [12]. However, we-disease appraisal may fluctuate daily, especially for an illness such as HIV that involves a difficult daily regimen (e.g., taking ART as prescribed). We-disease appraisal could fluctuate day-to-day as partners are perceived as more or less in collaborative ways. Furthermore, we-disease appraisal may fluctuate for some individuals but operate as an individual difference variable for others [13]. Only a few studies have examined both within-person (WP) and between-person (BP) levels of we-disease appraisal [13, 14].

## Common dyadic coping

The systemic transactional model [9, 15] posits that dyadic coping behaviors are expressed through supportive responses to a partner’s stress (supportive dyadic coping), the transfer of one’s responsibilities to the partner (delegated dyadic coping), and joint efforts to manage stress (common dyadic coping, common dyadic coping). Common dyadic coping has been reported to be more effective for couples coping with chronic illness than other dyadic coping strategies [16–18]. In a meta-analysis on couples facing cancer, common dyadic coping was a stronger predictor of relationship satisfaction (*r* = 0.42) than other dyadic coping behaviors (*r* = 0.17 to 0.34) [19]. This aligns with the systemic transactional model [9, 15], which anticipates common dyadic coping in situations where the stressor impacts both partners similarly and simultaneously, creating a “we-stress” situation. Serious medical conditions typically represent a shared experience of demands, as both partners are affected by the illness, creating a sense of a “we-disease” [20]. Therefore, common dyadic coping may be particularly crucial when a male couple is coping with HIV, a situation shared by both partners [4, 21].

## Actor–partner interdependence model and common fate model

Two prominent frameworks for analyzing dyadic data are the actor–partner interdependence model (APIM) [22] and common fate model (CFM) [23]. While both frameworks address dyadic data analysis, they differ in their theoretical foundations, analytical approaches, and variable considerations.

The APIM emphasizes interpersonal effects, examining how an individual’s characteristics affect both their own outcomes (actor effects) and their partner’s outcomes (partner effects), making it valuable for studying support exchanges in the patient–caregiver dyad through self-referential measures [24–26].

Contrastingly, the CFM conceptualizes the dyad as a collective unit, focusing on the individual’s and their partner’s shared experiences that simultaneously affect both of them, in addition to emphasizing relationship-level constructs [27, 28].

The present study employs the CFM for three compelling reasons. First, we-disease appraisal and common dyadic coping are inherently dyad-level constructs, co-constructed and agreed upon by both partners. Second, these constructs manifest as single latent variables that generate responses from both partners. Third, while APIM-based daily diary studies are abundant, no research has employed the CFM to examine how daily shared experiences affect both partners’ relational and individual well-being. To date, only one cross-sectional study of serodiscordant couples has adopted the CFM to assess the dyadic coping process [18]. While research has indicated that dyadic coping processes may fluctuate daily [13, 29, 30], no studies have investigated the shared experience of such fluctuations. Furthermore, existing dyadic daily diary research on dyadic coping has focused exclusively on couples managing noncommunicable conditions like cancer, chronic pain, and diabetes. The dyadic coping process among HIV serodiscordant couples presents unique challenges that distinguish it from the process for other chronic illnesses where one partner primarily assumes a caregiving role.

## Daily diary in HIV context

While it is challenging to apply the daily diary approach to HIV serodiscordant male couples, evidence from individual-level studies has demonstrated the value of this methodology for understanding how PLWHs cope with HIV-related stressors (e.g., stigma). The daily diary approach also offers distinct advantages over traditional questionnaires since it allows for capturing real-time variations in stigma experiences, minimizing recall bias, and enhancing ecological validity [31]. Studies have shown that daily experiences of HIV-related stigma predict worsened emotional well-being [32], with social support potentially buffering these negative effects [33]. Since HIV-related stigma can affect both PLWHs and their seronegative partners in daily life, a daily diary design would provide valuable insights into how serodiscordant couples collectively cope with HIV-related challenges.

## Chinese context of dyadic coping in serodiscordant couples

Serodiscordant male couples in China encounter distinct contextual factors that influence their coping mechanisms. First, the collectivist nature of Chinese culture may contribute to the perpetuation of stigma, as it emphasizes a social pattern characterized by strong interpersonal connections, a sense of belonging to a cohesive in-group, social harmony and conformity [34]. Research has consistently shown that Chinese societies exhibit an exceptionally high level of stigma, particularly in the context of HIV and sex minority [35–37]. Consequently, serodiscordant male couples frequently struggle to access coping resources beyond their intimate relationships. Second, family dynamics in Chinese culture play a central role, with strong expectations regarding marriage and procreation. As a result, such couples may experience pressure to conform to traditional family roles, complicating their ability to disclose their same-sex relationship and HIV status as they may feel compelled to conceal their relationship from family members, further limiting their access to familial support. Third, in heterosexual relationships, traditional masculinity norms often inhibit emotional expression and open communication. This cultural emphasis can make it challenging for partners to engage in effective dyadic coping strategies, as men may feel pressured to conform to societal expectations of stoicism and emotional restraint. In contrast, same-sex male couples may experience different dynamics in their dyadic coping processes. While they also face societal stigma related to both their sexual orientation and HIV status, the absence of traditional gender roles can create a more egalitarian space for emotional expression and communication [38]. Given these complexities, this study aims to deepen our understanding of the dyadic coping processes among serodiscordant male couples in China.

## Present study

**Fig. 1:**
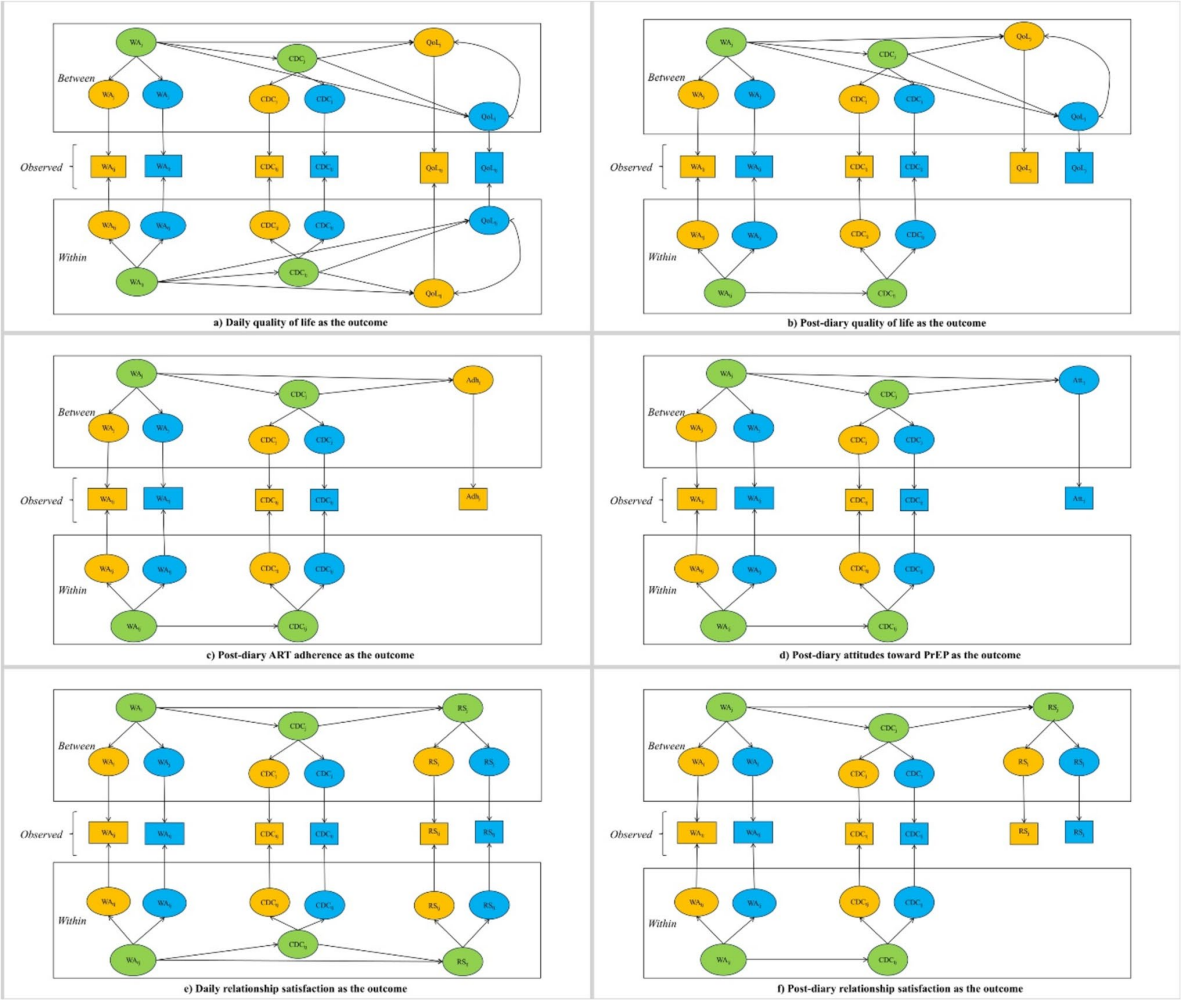
The Hypothetical Multilevel Common Fate Mediation Model *Adh* ART adherence, *Att* Attitudes toward PrEP, *WA* We-disease appraisal, *CDC* Common dyadic coping, *QoL* Quality of life; *RS* Relationship satisfaction, Subscript (_*j*_) indicates cluster; Subscript (_*t*_) indicates time. The variables for PLWHs, their partners, and dyads were marked yellow, blue, and green, respectively

Drawing from the dyadic coping models [9, 10, 15] and using a dyadic daily diary study design, we aimed to examine the connections between we-disease appraisal and individual outcomes—quality of life, ART adherence, and pre-exposure prophylaxis attitude (PrEP)—as well as relational outcomes like relationship satisfaction among Chinese serodiscordant male couples. As HIV has transitioned from a fatal illness to a manageable chronic condition, prioritizing the quality of life for PLWHs has become increasingly vital. The proposed “fourth 90” treatment goal underscores this focus, extending the continuum-of-care model beyond immune reconstitution and viral suppression [39]. This shift necessitates an emphasis on quality of life in our study. In serodiscordant couples, ART adherence and PrEP attitude are crucial for preventing cross-partner HIV transmission. Therefore, we choose ART adherence and PrEP attitude as individual outcomes. Furthermore, relationship satisfaction is strongly associated with individual health [40] and risk-taking behaviors [41], thus identifying predictors of relationship satisfaction and has important clinical implications not only for relational health, but also for individual health. We also assess the mediating role of common dyadic coping in these associations. Figure 1 shows our hypothetical models.

## Materials and methods

### Participants

We considered couples to be eligible for the study if they met the following criteria: (a) Both partners were at least 18 years old, (b) both partners were male, (c) the couple had been in a romantic relationship for a minimum of six months, (d) the couple was serodiscordant (i.e., one partner was HIV-positive and the other was HIV-negative), (e) both partners had daily access to a smartphone or computer for completing assessments, and (f) both partners were willing to participate. We excluded couples from the study if either partner (a) could not complete the daily assessments due to low literacy levels or physical or psychological constraints, (b) had been diagnosed with another life-threatening disease such as cancer, or (c) anticipated any extended period (> 1 week) of separation during the study period. We recruited our sample via local grassroots organizations from seven major cities (i.e., Beijing, Changsha, Chengdu, Guangzhou, Hohhot, Shenzhen, and Xiamen) between June and September 2023. The recruitment methods included online posting, peer referrals, and flyers. Trained site staff screened all participants for eligibility, and participants provided informed consent to the site staff. Of the 173 couples that site staff reached, 20 (11.6%) declined to participate because one partner refused to consent, four (2.3%) were not eligible because their relationship duration was less than six months, and eight (4.6%) only completed the baseline assessment. The final analytical sample thus comprised 141 eligible couples. The 14-day survey completion rate for the couples was 95.6% (3,774/3,948). The Institutional Review Board of City University of Hong Kong approved this study. Informed consent was obtained from all individual participants included in the study. The process of study implementation is shown in Appendix.

## Measures

### Pre-diary assessment

Before the daily diary assessment, participants provided information on demographics (i.e., age, ethnicity, educational attainment, sexual orientation, and personal monthly income), relationship characteristics (i.e., relationship length and marital status), and clinical information (e.g., self-reported viral load, CD4 + cell counts, years since diagnosis, and HIV disclosure).

### 14-day daily diary assessment

We measured we-disease appraisal, common dyadic coping, and quality of life, and relationship satisfaction during the diary phase. The sample item and scoring for the measures used is shown in Appendix.

**Daily We-Disease Appraisal.** We measured we-disease appraisal using a modified Inclusion of Other Scale in our previous study [18, 42]. In the present study, we tailored the introduction by adding a timeframe (i.e., today). This scale was rated on a seven-point scale (0–6). Higher reported scores indicated higher daily we-disease appraisal.

**Daily Common Dyadic Coping.** We measured common dyadic coping using a modified five-item subscale of the Dyadic Coping Inventory [43, 44]. The statements were assessed on a seven-point Likert scale (0–30). Higher reported scores indicated higher daily common dyadic coping. The Cronbach α ranged from 0.90 to 0.92 for PLWHs and 0.89 to 0.92 for partners across 14 days.

**Daily Quality of Life.** We measured quality of life using a modified single item [45; 46]. This item was rated on a seven-point Likert scale (0–6). Higher reported scores indicated higher daily quality of life. Siebens et al. [45] provided psychometric evidence for using the single-item quality of life scale in research and clinical care.

**Daily Relationship Satisfaction.** We measured relationship satisfaction using a modified single item [47]. This item was assessed on a seven-point Likert scale (0–6). Higher scores indicated higher daily relationship satisfaction. Niehuis et al. [47] found that the single-item measure of relationship satisfaction exhibited good psychometric properties in terms of test–retest reliability and convergent, concurrent, and predictive validity.

### Post-diary assessment

**Quality of Life.** Quality of life was measured using two versions of the World Health Organization Quality of Life questionnaire [48, 49]. For PLWHs, we used the 29-item brief version specifically designed for them (WHOQoL-HIV BREF, 0–116). For their partners, we used the brief version of the general WHOQoL questionnaire (WHOQoL BREF, 24 items, 0–96). Higher scores indicated a higher quality of life. The Cronbach α was 0.88 for PLWHs and 0.93 for their partners.

**PrEP Attitude.** PrEP attitude was measured using a five-item PrEP Attitude Scale [50]. We calculated the total score (0–20), with higher scores indicating more positive PrEP attitude. The Cronbach α was 0.85 for partners.

**ART Adherence.** ART adherence was assessed using a single question: “In the past 30 days, how many days did you take your HIV medication as directed? (0–30)”. Given that a standard of 95% adherence is frequently referenced as essential for achieving viral suppression [e.g., 51], we categorized individuals who took ART for more than 28 days as adherent, while those who did not were classified as non-adherent.

**Relationship Satisfaction.** Relationship satisfaction was measured using a four-item Couple Satisfaction Index [52, 53]. This scale was used in our previous study among Chinese serodiscordant couples [18]. We calculated the total score (0–20), with higher scores indicating better relationship satisfaction. The Cronbach α was 0.93 for PLWHs and 0.94 for partners.

## Statistical analysis

We used dyadic multilevel structural equation modeling to fit the common fate mediation model at both levels [27, 54] and used Monte Carlo method procedures for the confidence intervals of the indirect effect when managing nested data [55]. For more, we provided statistical details for mediation analyses in Appendix. In the present study, we modeled we-disease appraisal, common dyadic coping, and relationship satisfaction as common fate constructs, with quality of life, ART adherence, and PrEP attitude as noncommon fate constructs. Missing data were handled with full information maximum likelihood estimation. Analyses control for age, ethnicity, education level, sex orientation, personal monthly income, relationship length, self-reported viral load, CD4 + cell counts, and years since HIV diagnosis. All analyses were conducted using M*plus* version 8.

## Results

### Sociodemographic and clinical characteristics

On average, PLWHs were aged 30.80 (*SD* = 8.12, Table 1) and their partners were aged 30.66 (*SD* = 7.39). Most of the participants were of Han ethnicity (PLWHs, 86.5%; partners, 85.8%). Additionally, 63.1% of PLWHs and 68.8% of partners had obtained a bachelor’s degree or higher. Notably, 91.5% of PLWHs and 88.7% of partners self-identified as gay. Approximately half of the participants had a personal monthly income exceeding 5,000 CNY (PLWHs, 47.4%; partners, 47.5%). The average duration of the couple relationships was 3.53 years (*SD* = 3.47). A small proportion of participants had a heterosexual marriage (PLWHs, 5.7%; partners, 9.2%). Of all, 72.3% of PLWHs had an undetectable viral load, indicating successful treatment. Furthermore, 64.5% of PLWHs experienced immune function recovery. PLWHs had been diagnosed with HIV for 4.38 years on average. Interestingly, 52.5% of PLWHs disclosed their HIV status to their partners before entering a committed relationship. The ICC values for we-disease appraisal were 0.83 for both partners. For common dyadic coping, the values were 0.68 for PLWHs and 0.70 for their partners, while for quality of life, they were 0.58 and 0.60. Lastly, the ICC values for relationship satisfaction were 0.58 and 0.64, respectively (Table 2).

**Table 1:** Sample characteristics of Chinese HIV serodiscordant male couples (N = 141)

Variable	PLWHs n (%)	Partners n (%)
Demographics		
Age in years, mean (SD)	30.80 (8.12)	30.66 (7.39)
Ethnicity		
Han	122 (86.5)	121 (85.8)
Non-han	19 (13.5)	20 (14.2)
Education level		
Secondary school or below	19 (13.5)	11 (7.8)
High school	33 (23.4)	33 (23.4)
Bachelor degree	84 (59.6)	89 (63.1)
Master degree or above	5 (3.5)	8 (5.7)
Sexual orientation		
Gay	129 (91.5)	125 (88.7)
Bisexual	9 (6.4)	13 (9.2)
Other men who have sex with men	3 (2.1)	3 (2.1)
Personal monthly income, CNY		
No income	7 (5.0)	7 (5.0)
< 1,999	3 (2.1)	5 (3.5)
2,000–2,999	16 (11.3)	16 (11.3)
3,000–4,999	48 (34.0)	46 (32.6)
5,000–9,999	48 (34.0)	57 (40.4)
10,000–19,999	15 (10.6)	7 (5.0)
≥ 20,000	4 (2.8)	3 (2.1)
Relationship duration in years, mean (SD)	3.53 (3.47)	–
Marital status		
Married	8 (5.7)	13 (9.2)
Divorced	8 (5.7)	12 (8.5)
Never married	125 (88.7)	116 (82.3)
Clinical characteristics		
Self-reported Viral load		
Undetectable	102 (72.3)	–
Detectable	29 (20.6)	–
Unknown	10 (7.1)	–
Self-reported CD4, cells/ml		
< 200	4 (2.8)	–
350–499	39 (27.7)	–
≥ 500	91 (64.5)	–
Unknown	7 (5.0)	–
Years since diagnosis, mean (SD)	4.38 (3.35)	–
HIV disclosure		
Prior to the relationship	74 (52.5)	–
In relationship	67 (47.5)	–

PLWH: people living with HIV; SD: standardized deviation; CNY: Chinese Yuan

**Table 2:** Mean, Standard Deviation, Intra-class Correlation Coefficient for Study Variables

Variable	PLWHs			Partners			*r*_within_	*r*_between_
	*M*	*SD*	*ICC*	*M*	*SD*	*ICC*		
Daily diary assessment								
We-disease appraisal	4.04	1.81	83%	4.14	1.66	83%	.15	.44
Common dyadic coping	17.32	8.14	68%	17.78	7.42	70%	.27	.40
Quality of life^†^	4.39	1.34	58%	4.41	1.30	60%	.20	.43
Relationship satisfaction^‡^	4.42	1.43	58%	4.39	1.33	64%	.24	.44
Post-diary assessment								
Quality of life^†^	67.06	16.33	**–**	56.33	16.50	**–**	**–**	**–**
ART adherence^§^	28.9	3.64	**–**	**–**	**–**	**–**	**–**	**–**
PrEP attitude	**–**	**–**	**–**	14.77	3.98	**–**	**–**	**–**
Relationship satisfaction^‡^	14.06	4.14	**–**	14.16	3.94	**–**	**–**	**–**

M: mean; SD: standardized deviation; ICC: intraclass correlation coefficient

^†^Regarding quality of life, the same single-item scale was used for both partners during daily diary assessment; However, WHOQOL-BREF HIV scale was used for PLWHs and regular WHOQOL-BREF scale was used for HIV-negative partners during the post-diary assessment. The scores of PLWHs and their partners could not be compared as PLWHs completed the which contains an additional five items specific to HIV, while partners completed the regular WHOQOL-BREF scale. ^‡^Regarding relationship satisfaction, the same single-item scale was used during daily diary assessment; Both partners completed the four-item Couple Satisfaction Index during the post-diary assessment. ^§^Given that a standard of 95% adherence is frequently referenced as essential for achieving HIV viral suppression, we categorized individuals who took ART for more than 28 days as adherent (21/141, 14.9%), while those who did not were classified as non-adherent (120/141, 85.1%)

## Model for quality of life

**Fig. 2:**
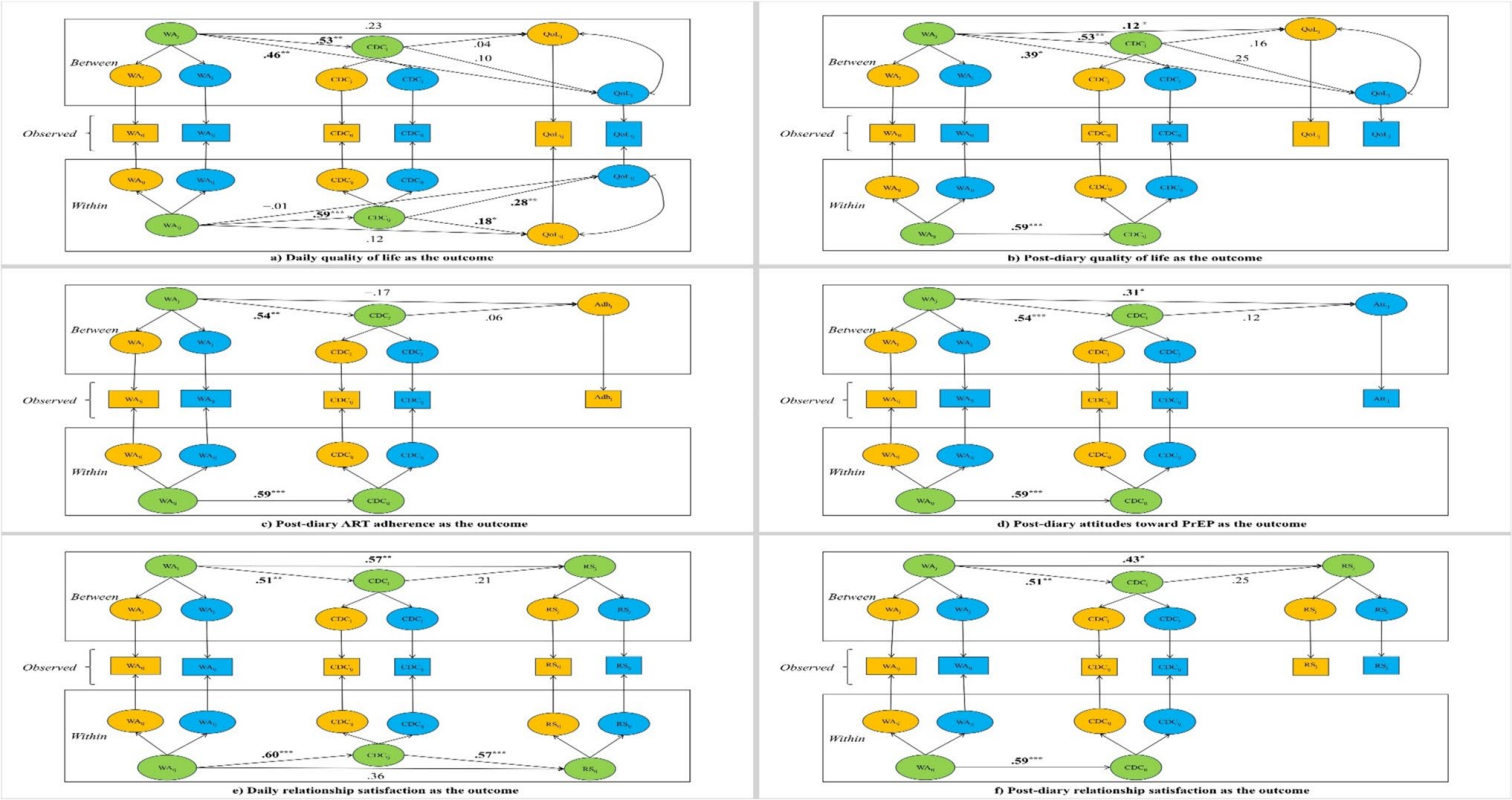
The Standardized Estimates for Each Pathway in Multilevel Common Fate Mediation Model *Adh.* ART adherence, *Att.* Attitudes toward PrEP, *WA* We-disease appraisal, *CDC* Common dyadic coping, *QoL* Quality of life, *RS* Relationship satisfaction, Subscript (_j_) indicates cluster; Subscript (_t_) indicates time. The variables for PLWHs, their partners, and dyads were marked yellow, blue, and green, respectively

As shown in Table 3 Model 1 and Fig. 2 Panel a, regarding WP level, on days when couples reported higher we-disease appraisal, they reported higher common dyadic coping (*b* = 4.78, *SE* = 1.15, *p* < 0.001). Furthermore, on days when common dyadic coping was higher, the couples reported higher quality of life (PLWHs: *b* = 0.07, *SE* = 0.03, *p* = 0.03; partners: *b* = 0.10, *SE* = 0.04, *p* = 0.008). However, neither direct effect of we-disease appraisal was significant for the PLWHs’ quality of life (*b* = 0.37, *SE* = 0.33, *p* = 0.25) or partners’ quality of life (*b* =  − 0.02, *SE* = 0.41, *p* = 0.95). Common dyadic coping significantly mediated the associations (PLWHs: *est* = 0.33, *SE* = 0.16, 95% *CI* [0.03, 0.68]; partners: *est* = 0.49, *SE* = 0.24, 95% *CI* [0.14, 0.99]), indicating that for every one-point increase in daily we-disease appraisal (on a 7-point Likert scale), the quality of life for PLWHs is expected to improve by 0.33 points. Similarly, for each one-point rise in daily we-disease appraisal, partners are expected to experience a 0.49-point increase in their quality of life, when considering only the indirect influence of common dyadic coping.

**Table 3:** Parameter estimates for multilevel SEM of outcomes as a function of daily we-disease appraisal and daily common dyadic coping

Parameter	*b*	*SE*	*β*	*t*	*p*	95% *CI*	
						*LL*	*UL*
Model 1: Daily Quality of Life							
Level 1 (within-person)							
WDA_PLWH_ → WDA_shared_	**–**	**–**	.38	**–**	**–**	**–**	**–**
WDA_partner_ → WDA_shared_	**–**	**–**	.41	**–**	**–**	**–**	**–**
CDC_PLWH_ → CDC_shared_	**–**	**–**	.49	**–**	**–**	**–**	**–**
CDC_partner_ → CDC_shared_	**–**	**–**	.56	**–**	**–**	**–**	**–**
WDA_shared_ → CDC_shared_	4.78^***^	1.15	.59	4.16	< .001	**–**	**–**
CDC_shared_ → QoL_PLWH_	0.07^*^	0.03	.18	2.17	.03	**–**	**–**
CDC_shared_ → QoL_partner_	0.10^**^	0.04	.28	2.67	.008	**–**	**–**
WDA_shared_ → QoL_PLWH_	0.37	0.33	.12	1.14	.25	**–**	**–**
WDA_shared_ → QoL_partner_	− 0.02	0.41	− .01	− 0.06	.95	**–**	**–**
**WDA**_**shared**_** → CDC**_**shared**_** → QoL**_**PLWH**_	0.33^*^	**0.16**	**–**	**2.03**	**.04**	**0.03**	**0.68**
**WDA**_**shared**_** → CDC**_**shared**_** → QoL**_**partner**_	0.49^*^	**0.24**	**–**	**2.01**	**.04**	**0.14**	**0.99**
Level 2 (between-person)							
WDA_PLWH_ → WDA_shared_	**–**	**–**	.63	**–**	**–**	**–**	**–**
WDA_partner_ → WDA_shared_	**–**	**–**	.69	**–**	**–**	**–**	**–**
CDC_PLWH_ → CDC_shared_	**–**	**–**	.61	**–**	**–**	**–**	**–**
CDC_partner_ → CDC_shared_	**–**	**–**	.65	**–**	**–**	**–**	**–**
WDA_shared_ → CDC_shared_	2.08^**^	0.60	.53	3.46	.001	**–**	**–**
CDC_shared_ → QoL_PLWH_	0.01	0.04	.04	0.25	.80	**–**	**–**
CDC_shared_ → QoL_partner_	0.02	0.05	.10	0.53	.59	**–**	**–**
WDA_shared_ → QoL_PLWH_	0.23^†^	0.14	.23	1.69	.09	**–**	**–**
WDA_shared_ → QoL_partner_	0.45^**^	0.17	.46	2.73	.006	**–**	**–**
WDA_shared_ → CDC_shared_ → QoL_PLWH_	0.02	0.08	**–**	0.25	.80	− 0.12	0.15
WDA_shared_ → CDC_shared_ → QoL_partner_	0.05	0.09	**–**	0.57	.57	− 0.14	0.25
Model 2: Post-diary Quality of Life							
Level 1 (within-person)							
WDA_PLWH_ → WDA_shared_	**–**	**–**	.37	**–**	**–**	**–**	**–**
WDA_partner_ → WDA_shared_	**–**	**–**	.41	**–**	**–**	**–**	**–**
CDC_PLWH_ → CDC_shared_	**–**	**–**	.49	**–**	**–**	**–**	**–**
CDC_partner_ → CDC_shared_	**–**	**–**	.56	**–**	**–**	**–**	**–**
WDA_shared_ → CDC_shared_	4.78^***^	1.15	.59	4.15	< .001	**–**	**–**
Level 2 (between-person)							
WDA_PLWH_ → WDA_shared_	**–**	**–**	.63	**–**	**–**	**–**	**–**
WDA_partner_ → WDA_shared_	**–**	**–**	.69	**–**	**–**	**–**	–
CDC_PLWH_ → CDC_shared_	**–**	**–**	.61	**–**	**–**	**–**	–
CDC_partner_ → CDC_shared_	**–**	**–**	.65	**–**	**–**	**–**	–
WDA_shared_ → CDC_shared_	2.08^**^	0.60	.53	3.46	.001	**–**	–
CDC_shared_ → QoL_PLWH_	0.65	0.56	.16	1.15	.25	**–**	–
CDC_shared_ → QoL_partner_	0.48	0.76	.25	0.64	.52	**–**	–
WDA_shared_ → QoL_PLWH_	3.84^*^	1.83	.12	2.10	.04	**–**	–
WDA_shared_ → QoL_partner_	6.20^*^	2.79	.39	2.23	.03	**–**	–
WDA_shared_ → CDC_shared_ → QoL_PLWH_	1.35	1.20	–	1.13	.26	− 0.98	4.00
WDA_shared_ → CDC_shared_ → QoL_partner_	1.01	1.51	–	0.67	.51	− 2.52	3.91
Model 3: Post-diary ART Adherence							
Level 1 (within-person)							
WDA_PLWH_ → WDA_shared_	–	–	.38	–	–	–	–
WDA_partner_ → WDA_shared_	–	–	.41	–	–	–	–
CDC_PLWH_ → CDC_shared_	–	–	.49	–	–	–	—

Parameter	*b*	*SE*	*β*	*t*	*p*	95% *CI*	
						*LL*	*UL*
CDC_partner_ → CDC_shared_	–	–	.56	–	–	–	–
WDA_shared_ → CDC_shared_	4.78^***^	1.15	.59	4.15	< .001	–	–
Level 2 (between-person)							
WDA_PLWH_ → WDA_shared_	–	–	.63	–	–	–	–
WDA_partner_ → WDA_shared_	–	–	.69	–	–	–	–
CDC_PLWH_ → CDC_shared_	–	–	.61	–	–	–	–
CDC_partner_ → CDC_shared_	–	–	.65	–	–	–	–
WDA_shared_ → CDC_shared_	2.10^**^	0.61	.54	3.44	.001	–	–
CDC_shared_ → Adh	0.03	0.10	.06	0.26	.80	–	–
WDA_shared_ → Adh	− 0.27	0.38	− .17	− 0.70	.48	–	–
WDA_shared_ → CDC_shared_ → Adh	0.06	0.22	—	0.26	.80	− 0.39	0.50
Model 4: Post-diary PrEP attitude							
Level 1 (within-person)							
WDA_PLWH_ → WDA_shared_	–	–	.37	–	–	–	–
WDA_partner_ → WDA_shared_	–	–	.41	–	–	–	–
CDC_PLWH_ → CDC_shared_	–	–	.49	–	–	–	–
CDC_partner_ → CDC_shared_	–	–	.56	–	–	–	–
WDA_shared_ → CDC_shared_	4.78^***^	1.15	.59	4.15	< .001	–	–
Level 2 (between-person)							
WDA_PLWH_ → WDA_shared_	–	–	.63	–	–	–	–
WDA_partner_ → WDA_shared_	–	–	.70	–	–	–	–
CDC_PLWH_ → CDC_shared_	–	–	.61	–	–	–	–
CDC_partner_ → CDC_shared_	–	–	.66	–	–	–	–
WDA_shared_ → CDC_shared_	2.09^***^	0.60	.54	3.49	< .001	–	–
CDC_shared_ → Att	0.11	0.15	.12	0.76	.45	–	–
WDA_shared_ → Att	1.17^*^	0.56	.31	2.10	.04	–	–
WDA_shared_ → CDC_shared_ → Att	0.24	0.28	–	0.83	.41	− 0.48	0.74
Model 5: Daily Relationship Satisfaction							
Level 1 (within-person)							
WDA_PLWH_ → WDA_shared_	–	–	.37	–	–	**–**	**–**
WDA_partner_ → WDA_shared_	–	–	.40	–	–	**–**	**–**
CDC_PLWH_ → CDC_shared_	–	–	.49	–	–	**–**	**–**
CDC_partner_ → CDC_shared_	–	–	.55	–	–	**–**	**–**
RS_PLWH_ → RS_shared_	–	–	.45	–	**–**	**–**	**–**
RS_partner_ → RS_shared_	**–**	**–**	.52	**–**	**–**	**–**	**–**
WDA_shared_ → CDC_shared_	4.90^***^	1.21	.60	4.05	< .001	**–**	**–**
CDC_shared_ → RS_shared_	0.11^***^	0.03	.57	3.67	< .001	**–**	**–**
WDA_shared_ → RS_shared_	0.54^†^	0.28	.36	1.93	.05	**–**	**–**
**WDA**_**shared**_** → CDC**_**shared**_** → RS**_**shared**_	0.52^**^	**0.17**	**–**	**3.00**	**.003**	**0.21**	**0.86**
Level 2 (between-person)							
WDA_PLWH_ → WDA_shared_	**–**	**–**	.64	**–**	**–**	**–**	**–**
WDA_partner_ → WDA_shared_	**–**	**–**	.69	**–**	**–**	**–**	**–**
CDC_PLWH_ → CDC_shared_	**–**	**–**	.59	**–**	**–**	**–**	**–**
CDC_partner_ → CDC_shared_	**–**	**–**	.64	**–**	**–**	**–**	**–**
RS_PLWH_ → RS_shared_	**–**	**–**	.61	**–**	**–**	**–**	**–**
RS_partner_ → RS_shared_	**–**	**–**	.64	**–**	**–**	**–**	**–**
WDA_shared_ → CDC_shared_	1.92^**^	0.57	.51	3.41	.001	**–**	**–**
CDC_shared_ → RS_shared_	0.04	0.03	.21	1.06	.29	**–**	**–**
WDA_shared_ → RS_shared_	0.37^**^	0.12	.57	3.18	.001	**–**	**–**
WDA_shared_ → CDC_shared_ → RS_shared_	0.07	0.06	**–**	1.13	.26	− 0.06	0.18

Parameter	*b*	*SE*	*β*	*t*	*p*	95% *CI*	
						*LL*	*UL*
Model 6: Post-diary Relationship Satisfaction							
Level 1 (within-person)							
WDA_PLWH_ → WDA_shared_	**–**	**–**	.38	**–**	**–**	**–**	**–**
WDA_partner_ → WDA_shared_	**–**	**–**	.41	**–**	**–**	**–**	**–**
CDC_PLWH_ → CDC_shared_	**–**	**–**	.49	**–**	**–**	**–**	**–**
CDC_partner_ → CDC_shared_	**–**	**–**	.55	**–**	**–**	**–**	**–**
WDA_shared_ → CDC_shared_	4.77^***^	1.15	.59	4.16	< .001	2.53	7.02
Level 2 (Between-person)							
WDA_PLWH_ → WDA_shared_	**–**	**–**	.64	**–**	**–**	**–**	**–**
WDA_partner_ → WDA_shared_	**–**	**–**	.69	**–**	**–**	**–**	**–**
CDC_PLWH_ → CDC_shared_	**–**	**–**	.61	**–**	**–**	**–**	**–**
CDC_partner_ → CDC_shared_	**–**	**–**	.66	**–**	**–**	**–**	**–**
RS_PLWH_ → RS_shared_	**–**	**–**	.60	**–**	**–**	**–**	**–**
RS_partner_ → RS_shared_	**–**	**–**	.63	**–**	**–**	**–**	**–**
WDA_shared_ → CDC_shared_	2.01^**^	0.59	.51	3.40	.001	**–**	**–**
CDC_shared_ → RS_shared_	0.15	0.13	.25	1.19	.23	**–**	**–**
WDA_shared_ → RS_shared_	1.06^*^	0.44	.43	2.27	.02	**–**	**–**
WDA_shared_ → CDC_shared_ → RS_shared_	0.31	0.26	**–**	1.21	.23	− 0.23	0.85

Bolded pathways indicate significant mediating effects where the confidence interval does not include zero

WDA: we-disease appraisal; CDC: common dyadic coping; QoL: quality of life; RS: relationship satisfaction; *b*: unstandardized estimates; *SE*: standard error; *β*: standardized estimates; *CI*: confidence interval; *LL*: lower limit; *UL*: upper limit. We calculated Monte Carlo CI for mediation effects. The model fit was satisfactory (**Model 1**: CFI = .94, TLI = .96, RMSEA = .01, SRMR_within_ = .01, SR MR_between_ = .09; **Model 2**: CFI = .91, TLI = .87, RMSEA = .02, SRMR_within_ = .01, SRMR_between_ = .09; **Model 3**: Not available for binary outcome; **Model 4**: CFI = .96, TLI = .95, RMSEA = .01, SRMR_within_ = .01, SRMR_between_ = .08; **Model 5**: CFI = .98, TLI = .97, RMSEA = .01, SRMR_within_ = .01, SRMR_between_ = .09; **Model 6**: CFI = .94, TLI = .91, RMSEA = .01, SRMR_within_ = .01, SRMR_between_ = .09). Significant mediation pathways were bolded. ^†^*p* < .1 ^*^p < .05 ^**^p < .01.^***^p < .01

Regarding BP level, when the average we-disease appraisal was higher, couples reported higher common dyadic coping (*b* = 2.08, *SE* = 0.60, *p* = 0.001). However, no significant association existed between common dyadic coping and PLWHs’ quality of life (*b* = 0.01, *SE* = 0.04, *p* = 0.80) or partners’ quality of life (*b* = 0.02, *SE* = 0.05, *p* = 0.59). Regarding direct effects, we-disease appraisal was associated with higher quality of life at a trend level for PLWHs and significant level for partners (PLWHs: *b* = 0.23, *SE* = 0.14, *p* = 0.09; partners: *b* = 0.45, *SE* = 0.17, *p* = 0.006). Common dyadic coping did not mediate the association between we-disease appraisal and both partners’ quality of life (PLWHs: *est* = 0.02, *SE* = 0.08, 95% *CI* [− 0.12, 0.15]; partners: *est* = 0.05, *SE* = 0.09, 95% *CI* [− 0.14, 0.25]), indicating that, on average, for each one-point increase in we-disease appraisal (on a 7-point Likert scale), the quality of life for PLWHs is expected to increase by only 0.02 points. Similarly, partners are expected to experience an increase of 0.05 points in their quality of life for each one-point rise in we-disease appraisal, when only considering the indirect influence of common dyadic coping.

Regarding post-diary quality of life (Table 3, Model 2; Fig. 2 Panel b), common dyadic coping cannot predict both partners’ post-diary quality of life (PLWHs: *b* = 0.65, *SE* = 0.56, *p* = 0.25; Partners: *b* = 0.48, *SE* = 0.76, *p* = 0.52, Table 3 Panel B). However, we-disease appraisal significantly predicted both partners’ post-diary quality of life (PLWHs: *b* = 3.84, *SE* = 1.83, *p* = 0.04; Partners: *b* = 6.20, *SE* = 2.79, *p* = 0.03). Moreover, we failed to find the mediating effect of common dyadic coping (PLWHs: *est* = 1.35, *SE* = 1.20, 95% *CI* [− 0.98, 4.00]; Partners: *est* = 1.01, *SE* = 1.51, 95% *CI* [− 2.52, 3.91]), indicating that, on average, for every one-point increase in we-disease appraisal during the daily diary (on a 7-point Likert scale), the quality of life for PLWHs is expected to improve by 1.35 points in the follow-up assessment. Similarly, partners are predicted to experience a 1.01-point increase in their quality of life in the follow-up assessment for each one-point rise in we-disease appraisal during the daily diary, considering only the indirect influence of common dyadic coping.

## Model for ART adherence and PrEP attitude

As shown in Table 3, Model 4 and Fig. 2 Panel c, our analyses did not find significant associations between we-disease appraisal and ART adherence (*b* =  − 0.27, *SE* = 0.38, *p* = 0.48), common dyadic coping and ART adherence (*b* = 0.03, *SE* = 0.10, *p* = 0.80) or evidence of the mediating effect of common dyadic coping (*est* = 0.06, *SE* = 0.22, 95% *CI* [− 0.39, 0.50]), indicating that for every one-point increase in we-disease appraisal, on average, during the daily diary (on a 7-point Likert scale), ART adherence is expected to only increase by 0.06 points in the follow-up assessment, considering only the indirect influence of common dyadic coping. Moreover, as shown in Table 3, Model 5 and Fig. 2 Panel d, common dyadic coping did not predict the PrEP attitude (*b* = 0.11, *SE* = 0.15, *p* = 0.45) or mediate between we-disease appraisal and PrEP attitude (*est* = 0.24, *SE* = 0.28, 95% *CI* [− 0.48, 0.74]), implying that for every one-point increase in we-disease appraisal, on average, during the daily diary (on a 7-point Likert scale), PrEP attitude is expected to increase by 0.24 points in the follow-up assessment, considering only the indirect influence of common dyadic coping. However, we-disease appraisal significantly predicted PrEP attitude (*b* = 1.17, *SE* = 0.56, *p* = 0.04).

## Model for daily relationship satisfaction

As shown in Table 3, Model 5 and Fig. 2 Panel e, regarding WP level, when couples reported higher we-disease appraisal, they reported higher common dyadic coping (*b* = 4.90, *SE* = 1.21, *p* < 0.001, Table 3 Panel E). Furthermore, when common dyadic coping was more prevalent, couples reported higher relationship satisfaction (*b* = 0.11, *SE* = 0.03, *p* < 0.001). Regarding the direct effect, on days in which we-disease appraisal was higher, couples reported higher relationship satisfaction (*b* = 0.54, *SE* = 0.28, *p* = 0.05). Our analyses also showed that common dyadic coping acted as a mediator in this association (*est* = 0.52, *SE* = 0.17, 95% *CI* [0.21, 0.86]), indicating that for every one-point increase in daily we-disease appraisal (on a 7-point Likert scale), couples’ relationship satisfaction is expected to improve by 0.52 points, when considering only the indirect influence of common dyadic coping.

Regarding BP level, when the average we-disease appraisal was higher, couples reported higher common dyadic coping (*b* = 1.92, *SE* = 0.57, *p* = 0.001). However, common dyadic coping was not related to relationship satisfaction (*b* = 0.04, *SE* = 0.03, *p* = 0.29). Regarding the direct effect, when the average we-disease appraisal was higher, couples reported higher relationship satisfaction (*b* = 0.37, *SE* = 0.12, *p* = 0.001). We found no evidence of the mediating effect of common dyadic coping (*est* = 0.07, *SE* = 0.06, 95% *CI* [− 0.06, 0.18]), indicating that for every one-point increase in average we-disease appraisal (on a 7-point Likert scale), couples’ relationship satisfaction is expected to only increase by 0.07 points on average, when considering only the indirect influence of common dyadic coping.

As shown in Table 3, Model 6 and Fig. 2 Panel f, regarding post-diary relationship satisfaction, there was a positive association between we-disease appraisal and post-diary relationship satisfaction (*b* = 1.06, *SE* = 0.44, *p* = 0.02). However, we found no significant association between common dyadic coping and post-diary relationship satisfaction (*b* = 0.15, *SE* = 0.13, *p* = 0.23) and no evidence of the mediating effect of common dyadic coping (*est* = 0.31, *SE* = 0.26, 95% *CI* [− 0.23, 0.85]), implying that for every one-point increase in we-disease appraisal in average during the daily diary (on a 7-point Likert scale), their relationship satisfaction is expected to improve by 0.31 points in the follow-up assessment, when considering only the indirect influence of common dyadic coping.

## Discussion

This initial study sought to understand WP variability in the dyadic coping process using CFM in Chinese serodiscordant male couples. The absence of obvious differences in PLWHs and their partners rate of we-disease appraisal and common dyadic coping suggests a more egalitarian dynamic within serodiscordant same-sex relationships, which replicated our earlier cross-sectional findings using the same measurement [18]. Our findings also support earlier reports of WP variability in the dyadic coping process [13; 25] by showing that the BP differences in we-disease appraisal and common dyadic coping exceeded the WP variations. This disparity suggests that the coping journey for couples managing HIV is prolonged, where day-to-day fluctuations are infrequent if no urgent stressors emerge. An alternative explanation for these findings is that these couples had established relatively stable dyadic coping mechanisms by the time of the study, given that the PLWHs had been diagnosed with HIV and had maintained relationships with their partners for several years. Although dyadic coping processes among serodiscordant couples are relatively stable, other data analyses with diabetes samples found that greater variability in we-disease appraisal in spouses is associated with less secure attachments and poorer diabetes outcomes [56]. Future work is required to understand whether variability in the dyadic coping process is associated with characteristics of the PLWHs, the partners, the relationship, or HIV and its associated daily stressors. For example, precious studies in couples in which one person had type one diabetes showed that greater variability in we-disease appraisal in spouses is related to less secure attachment [56].

## Common fate at both levels

Our analyses suggest that we-disease appraisal and common dyadic coping can be modeled as common fate variables at the WP level and BP level. Notably, the results indicate that both constructs at the BP level account for a greater variance, as evidenced by larger standardized factor scores for both partners compared to the WP level. Extending our previous cross-sectional study among serodiscordant couples using the common fate medication model [18], the present study highlighted that daily fluctuations in the dyadic coping process are intricately interlocked within couples. Our findings align with those of previous studies that have documented daily fluctuations in dyadic coping among couples managing type 1 diabetes [13] and type two diabetes [14]. We extend these findings by demonstrating that both partners’ responses tend to co-fluctuate, suggesting a truly dyadic process. Future work is needed to assess whether such co-fluctuation can be generalized to other dyadic constructs (e.g., co-rumination).

## Bivariate associations for we-disease appraisal and common dyadic coping as well as outcomes

While we-disease appraisal and common dyadic coping were linked at WP level and BP level, their associations with various outcomes differed. In general, daily we-disease appraisal was associated with relationship satisfaction. Conversely, average we-disease appraisal demonstrated a broader range of associations (better quality of life for partners during the diary period, better post-diary quality of life for both partners, more positive PrEP attitude among partners, greater relationship satisfaction both during the diary period and afterward). Moreover, daily common dyadic coping was associated with better quality of life for partners and greater relationship satisfaction during the diary period. Conversely, we failed to identify any significant associations for common dyadic coping at BP level. A shared appraisal may be related to these outcomes because individuals feel they are part of a team in managing HIV [18], or because both partners include each other’s stress as part of their own future goals [10] or because a we-disease appraisal may reduce secondary stress appraisals regarding possible coping resources [57]. In contrast, common dyadic coping consists of tangible actions, which may serve as an observable signal to couple members that they are working as a team to manage HIV in daily life, thereby leading to more immediate effects during the diary period.

## Mediating role of common dyadic coping

Common dyadic coping mediated the associations between we-disease appraisal and quality of life and relationship satisfaction at WP level. This dynamic relationship suggests an immediate and interconnected link between these variables from day to day. However, the mediation effects observed at the WP level were not replicated at the BP level. This discrepancy indicates that a couple’s traits or characteristics associated with common dyadic coping may not consistently predict the levels or changes in outcomes when comparing different dyads [58]. Stable dyad and contextual factors cannot explain the findings, as dyads show higher quality of life and relationship satisfaction on days when they have more common dyadic coping. Despite the absence of the mediating role of common dyadic coping at the BP level, BP differences in we-disease remain highly predictive of most outcomes, suggesting other possible mediating pathways may exist. For example, two qualitative dyadic studies consistently showed that disengaged avoidance occurred when couples appraised the stressor as an individual stress affecting each of them individually rather than as a couple [38; 59]. However, common negative coping was not included in the measure used in the present study. Therefore, future studies are required to expand the present study by considering the positive and negative aspects of common dyadic coping. While common dyadic coping represents a joint effort, with both partners working together to manage stress, other forms of dyadic coping, particularly supportive dyadic coping (where one partner assists the other), may also occur in couples’ daily lives. Daily diary studies examining partner support as a unidirectional form of dyadic coping have yielded mixed findings among PLWHs. Some studies have found that perceived emotional support helps to buffer the negative impact of stigma on emotional well-being, although this effect is limited to specific periods and contexts, particularly during heightened stress, such as occurred during the early COVID-19 pandemic [33]. Other studies have shown that only provided support (not received support) buffers the negative effect of HIV-related stress, but this buffering effect is only present among those in intimate relationships, while single participants often experience detrimental effects [60]. Importantly, these studies have primarily collected data from PLWHs alone, without considering their partners’ perspectives. Since partner support represents a transactional process requiring balance and reciprocity (as suggested by equity theory), future research should adopt dyadic approaches to better understand support exchange processes between both partners in serodiscordant couples.

Moreover, our findings were echoed by another study utilizing the APIM in a daily diary design, which demonstrates significant associations between daily shared illness appraisals and various relational and individual outcomes in couples managing diabetes [13]. This cited study further identified significant moderating effects of shared illness among certain outcomes, indicating that collaborative coping is beneficial only when the illness is perceived as a shared experience. Therefore, future research should examine daily dyadic interdependence as both a common fate and a partner influence, as well as the role of we-disease appraisal, which may function as either a starting point or a buffering factor in the dyadic coping process. Such investigations could significantly contribute to theoretical advancements in this field.

## Implications and limitations

Most of the research in this area has focused on the common dyadic coping strategies adopted by both partners rather than whether individuals view the illness as our-disease or my/your disease [61]. Our findings show that how an illness is appraised on a daily basis might shape the dyadic coping process. The benefits of a we-disease appraisal are especially important in the context of China as collectivist culture and traditional family dynamics may prevent such couples for seeking support outside their relationship. Both partners may benefit if they are able to share their illness within relationship by forming co-constructing the meaning of the illness and increasing co-involvement in HIV management. Although no clinical trials have specifically targeted the dyadic coping process in HIV settings, some couple-based interventions have shown promising effects on various outcomes—such as both partners’ disease appraisal, collaboration, and efficacy—among couples dealing with other chronic illnesses [62–64]. Moreover, dyadic coping-focused intervention may be effective with just one session [62], indicating that these interventions could be highly cost-effective in clinical settings. Our findings offer additional insights, suggesting that future intervention trials should place greater emphasis on co-fluctuation within the daily dyadic coping process as a shared experience.

While our study offers valuable insights, its limitations must be acknowledged. Despite our use of daily measurements to capture variable fluctuations, our study was constrained to PLWHs diagnosed for several years. Future investigations could focus on long-term WP changes by employing a measurement burst design, enabling the exploration of WP processes at various time intervals from new diagnosis to later stages. As our study used a daily diary approach, the measurement of some constructs was simplified to a single item, representing a significant limitation in the depth of assessment. However, considering the burden of daily diary study, we measured same construct using different measures during the diary and post-diary, which may limit the comparability of findings. Moreover, the present study only assessed PrEP attitudes rather than usage patterns and barriers to adoption, which may have limited our understanding of the complete PrEP uptake process among serodiscordant couples. Future studies should aim to incorporate both quantitative behavioral measures of PrEP usage and qualitative assessments of the barriers encountered during implementation. For example, researchers could assess other psychosocial factors such as accessibility, stigma, low perceived risk, and individual motivations, which may influence PrEP adoption and adherence. Another key limitation of this study is that the 2-month follow-up period may not have allowed us to fully capture the long-term evolution of couples’ coping strategies and relationship dynamics, particularly given the chronic nature of HIV management. Future studies should thus consider implementing longer follow-up periods (e.g., 6–12 months) to examine the temporal stability of dyadic coping patterns and their impact on outcomes among serodiscordant couples. Additionally, incorporating multiple follow-up assessments would help track the trajectory of adjustment processes over time. Furthermore, estimating these daily processes can inform macro-level changes that occur more slowly—over months, years, or even decades—and are considered more distal outcomes within a dynamic system [65].

## Conclusion

In summary, this study demonstrates that we-disease appraisal and common dyadic coping are differentially associated with individual and dyadic outcomes in serodiscordant male couples. Furthermore, evidence suggests a potential mediating role of common dyadic coping, particularly for daily quality of life and daily relationship satisfaction. These findings have significant implications for developing targeted interventions for couples navigating HIV-related challenges. Specifically, interventions that promote co-fluctuation in daily dyadic coping processes and establish stable dyadic coping patterns may enhance both partners’ adjustment to HIV.

## Supplementary Information

Below is the link to the electronic supplementary material.

Supplementary file1 (DOCX 15 kb)

## Data Availability

The data that support the findings of this study are available on request from the corresponding author (Dr. Nancy Xiaonan Yu) upon reasonable request.
