# Supplementary material for: Delineating the dyadic coping process in HIV serodiscordant male couples: a dyadic daily diary study using the common fate model
Source: Qual Life Res. 2025 Feb 3;34(5):1317–32. doi: 10.1007/s11136-025-03903-4 (PMC12064454; doi:10.1007/s11136-025-03903-4)
Supplement: Supplementary file 1 — Supplementary file1 (DOCX 15 kb) [file 11136_2025_3903_MOESM1_ESM.docx]

**Delineating the Dyadic Coping Process in HIV Serodiscordant Male Couples:**

**A Dyadic Daily Diary Study Using the Common Fate Model**

**Study Implementation**

Initially, PLWHs and their partners provided information on their sociodemographic and clinical characteristics. Subsequently, we collected web-based daily diaries for 14 consecutive days. Trained staff at the site instructed each participant to independently complete the diary every evening, with each entry taking approximately 5 minutes. We assigned a unique ID and QR code to each member of the couple and asked them to respond to the diary using their smartphones. We sent a reminder to all participants at 8:00 pm each evening to prompt them to complete the diary. If a participant did not respond by 10:00 pm, a follow-up reminder was sent to minimize the probability of missing entries. Two months after completing the daily diaries, all participants were invited to partake in a follow-up assessment. We collected all data via an online platform (i.e., Wenjuanxing, https://www.wjx.cn/). The couples received compensation of up to 420 CNY (i.e., 30 CNY/day *14 days = 420 CNY) for daily surveys and 180 CNY for pre-diary and post-diary assessments.

**Measurement Details**

For daily we-disease appraisal, we tailored the introduction by adding a timeframe (i.e., today): “One circle in each pair is labeled ‘self,’ and the other circle is labeled ‘partner.’ You choose one of the seven pairs to answer the following question, ‘When you think about HIV today, do you view it as ‘our problem’ (shared equally by you and your partner) or primarily your own or partner’s problem?’ The region where the two circles overlap represents the degree to which you appraise HIV as ‘our problem’ today.).” For daily common dyadic coping, the statements were assessed on a seven-point Likert scale (e.g., “Today, we try to cope with HIV-related stress together and search for ascertained solutions.”), ranging from 1 (“not at all”) to 7 (“always”). For daily quality of life, this item was rated on a seven-point Likert scale (i.e., “Taking everything into account today, please rate your overall quality of life today on the following seven-point scale.”), ranging from 1 (“very bad”) to 7 (“very good”). For daily relationship satisfaction, this item was assessed on a seven-point Likert scale (i.e., “How satisfied are you with your relationship today?”), ranging from 1 (“not at all”) to 7 (“very satisfied”).

**Statistical Details for Mediation Analyses**

The dyadic diary data had two sources of nonindependence: a) the nonindependence between the two dyad members and b) the nonindependence of observations within each dyad member. Our model implicitly partitions each observed Level-1 variable into latent within-person (WP) and between-person (WP) components. The mediation effect at the WP level is *a* (predictor-to-mediator) × *b* (mediator-to-outcome) random effects + covariance between random a and b paths, while at the BP level, it is *a* × *b*. As the random effects for *a* and *b* in our sample were too low to warrant calculation or could not be calculated due to a model convergence issue, the indirect effect was the product of the *a* and *b* paths.
